# The effect of Massa Medicata Fermentata on the cytokine secretion of colonic mucosa and visceral sensitivity in rats with IBS-D

**DOI:** 10.3389/fcimb.2025.1616796

**Published:** 2026-01-02

**Authors:** Zhaomeng Zhuang, Bin Lv, Yi Chen, Jiayuan Chen

**Affiliations:** 1 Department of Gastroenterology, Zhejiang Chinese Medical University Affiliated Wenzhou Hospital of Integrated Traditional Chinese and Western Medicine, Wenzhou, China; 2 Department of Gastroenterology, The First Affiliated Hospital of Zhejiang Chinese Medical University, Zhejiang Provincial Hospital of Traditional Chinese Medicine, Hangzhou, China

**Keywords:** cytokine imbalance, Flagellin, IBS-D, Massa Medicata Fermentata (MMF), visceral hypersensitivity

## Abstract

**Objective:**

To study the effect of MMF(Massa Medicata Fermentata)on the secretion of cytokines of colon mucosa and visceral sensitivity in rats with IBS-D(diarrhea-predominant irritable bowel syndrome).

**Methods:**

45 adult male SD rats (Spragua-dawley) were randomly divided into Normal control group (NC). IBS-D model group (Model); MMF intervention group (MMF). qPCR (quantitative real-time polymerase chain reaction) was used to detect the quantity of fecal *Bifidobacterium*, *Lactobacillus*, and *Escherichia coli*; FITC-Dextran (Fluorescein Isothiocyanate-Dextran) was used to detect intestinal permeability; Immunofluorescence was used to detect the localization of FliC (Flagellin main component) and TLR5 (Toll-like receptor 5) in the intestinal mucosa; qPCR and WB (Western blot) were used to detect the mRNA and protein expression of TLR5, TRIF (Toll/Interleukin-1 Receptor Domain-Containing Adapter Inducing Interferon-β), EPK1/2 (Extracellular signal-regulated kinase 1/2) in LPDCs (Lamina Propria Dendritic Cells); CCK8 (Cell Counting Kit-8) was used to detect the proliferation of CD4+ T lymphocytes stimulated by LPDCs; ELISA (Enzyme-Linked Immunosorbent Assay) was used to detect the secretion of IL-12, IL-4, IL-6, IL-9, IL-17A, IL-10 (Interleukin-12, 4, 6, 9, 10, 17A), IFN-γ (interferon-gamma), TGF-β (Transforming Growth Factor - beta), from CD4+ T lymphocytes.

**Results:**

After IBS-D modeling, When compared with the NC group, the visceral sensitivity of rats in model group and MMF group were increased; The quantities of fecal *Lactobacillus* and *Bifidobacterium* decreased, The quantities of fecal *Escherichia coli* increased; The permeability of colonic mucosa was enhanced, accompanied with Flagellin and TLR5 protein upregulated; The expression of TLR5, TRIF, EPK1/2 signals inside LPDCs were increased; CD4+ T lymphocytes proliferation ability was hyperfunction, followed by the excessive secretion of IL-6, IL-17A, TGF-β, IL-12, IFN-γ, and IL-4 from CD4+ T lymphocytes, in contrast IL-10 was hyposecretion (p<0.05 for all). While when compared with the Model group, the above situations have been found recovered in the MMF group. (p<0.05 for all).

**Conclusion:**

MMF can alleviate the abnormal immune response and cytokine secretion of colon and relieve visceral hypersensitivity symptoms in rats with IBS-D. Its target may be closely related to the FliC-TLR5-TRIF-ERK1/2 pathway.

## 1 Introduction

The primary pathology associated with diarrhea-predominant irritable bowel syndrome (IBS-D) is “visceral hypersensitivity” (Lacy et al., 2021; Shin and Kashyap, 2023). Many studies have shown that imbalance of intestinal microbiota and increased colonization of harmful bacteria, characterized by an increased abundance of *Escherichia coli* and a decreased abundance of *Bifidobacterium and Lactobacillus*, which was one of the important pathogenesis and clinical manifestations of IBS-D patients, which are considered to be the etiological factors of visceral sensitivity in IBS-D patients (Almonajjed et al., 2025), this was consistent with the results of our preliminary research (Zhuang et al., 2022).

The same as other researchers, we also have found that the infiltration of chronic inflammatory factors in the mucosa, initiated by the immune activation of intestinal mucosal lamina propria dendritic cells (LPDCs), which mediates an abnormal immune response in the intestinal mucosa, is a significant contributor to visceral hypersensitivity in IBS-D (Li et al., 2015; Zhuang et al., 2016; Yuan et al., 2023).

Through our previous studies, we found that there has an elevation of bacterial flagellin content in rats with IBS-D, accompanied by an upregulation of TLR5 and TRIF protein expression in the colonic mucosa, which is closely associated with their symptoms of visceral hypersensitivity (Zhuang et al., 2022). Through cell experiments we demonstrated that the complex signaling pathway involving FliC and Toll-like receptor 5 (TLR5) promotes the proliferation and maturation of the cellular phenotype of myeloid dendritic cells, which activated the TRIF-ERK1/2 pathway within the dendritic cells, resulting in the enhancement of their immune functions and an improved ability to present antigens, causing the alterations in the types of cytokines secreted by lymphocytes (Zhuang et al., 2024). Therefore, it is suggested that the bacterial flagellin of *Escherichia coli* may play a great role in the pathogenesis of IBS-D visceral hypersensitivity by mediating an imbalance in cytokine secretion via the FliC-TLR5-TRIF-p-ERK1/2 pathway of colon mucosa.

However, this pathway has not been verified *in vivo*, so the specific mechanism between excessive expression of flagellum protein caused by intestinal microbiota imbalance and abnormal immune response of intestinal mucosa and imbalance of cytokine secretion has not been elucidated.

The traditional Chinese medicine “Massa Medicata Fermentata(MMF)is a classic compound medicine naturally fermented from wheat flour, red adzuki beans, bitter apricot seeds, fresh Artemisia annua, fresh Polygonum hydropiper, and fresh Xanthium sibiricum seedlings, which is primarily used for allergic diarrhea, intestinal dysfunction-related diarrhea, and chronic diarrhea post infections, with particularly notable therapeutic effects (Sun et al., 2018; Zhang et al., 2020; Li et al., 2025). The results of our previous animal experiments suggested that MMF could alleviate the symptoms of visceral hypersensitivity in IBS-D rats, additionally, it could reduce the abundance of flagellated bacteria, such as *Escherichia coli*, while increasing the levels of *Lactobacillus* and *Bifidobacterium* in the feces of IBS-D rats, which led to a reduction in the content of flagellated proteins and the expression of TLR5 proteins in colon mucosa (Zhuang et al., 2022).

While the effectiveness of MMF on the permeability and cytokine secretion of colon mucosal has not been reported yet, meanwhile whether will MMF affects the FliC-TLR5-TRIF-p-ERK1/2 pathway by regulating gut microbiota remains to be further confirmed. Therefore, this experiment aimed to reveal the effect of MMF on the colon mucosa and visceral sensitivity of rats with IBS-D.

## 2 Materials and methods

### 2.1 Laboratory animals

A total of 45 adult male SD rats of SPF grade weighing 220~250g at 6–8 weeks of age were selected (produced and supplied by Chengdu Pharmachem Biotechnology Co.).

Animal license number: SCXK (Sichuan) 2020-034.

Animal Ethical Number: 2022-K006.

### 2.2 Instruments and reagents

#### 2.2.1 Main instruments

8F Foley catheter (2mm in diameter, maximum balloon capacity of 3mL, maximum diameter of 2cm), used as a rectal balloon dilation catheter (produced by Zhejiang Hengkang Medical Devices Co., Ltd); AMPure XP Beads (BECKMAN), Microplate reader (BioTek), MiSeq sequencer (Illumina), ELISA reader (Bio-Rad). Tanon-4200 Gel Imaging System (Tanon), Laser Scanning Confocal Fluorescence Microscope (Leica), Cell Magnetic Bead Sorting Rack (Miltenyi Biotec).

#### 2.2.2 Primary reagent

MMF (China Resources Sanjiu Co., Ltd); glacial acetic acid (10000208 Sinopharm, Inc.); FITC-Dextran (Sigma, Inc.); WB Antibodies: TLR5 Antibody (rabbit source, Proteintech, Inc.), TRIF Antibody (rabbit source, Affinity, Inc.); P-ERK1/2 Antibody (rabbit source, abcam, Inc.); immunofluorescent antibodies: immunofluorescent primary antibody: TLR5 (rabbit source, Three Eagles, Inc.), immunofluorescent primary antibody: Flic (mouse source, Yiqiao, Inc.), and Immunofluorescence secondary antibody: goat anti-rabbit IgGH&L (Cy3) (abcam, Inc.), immunofluorescence secondary antibody: goat anti-mouse IgGH&L (FITC) (abcam, Inc.); Immunohistochemistry antibody: immunohistochemistry primary antibody: TLR5 (rabbit source, proteintech, Inc.), immunohistochemistry primary antibody: TRIF (rabbit source, Affinity, Inc.), the Immunohistochemical primary antibody: p-ERK1/2 (rabbit source abcam), immunohistochemical secondary antibody: HRP-labeled goat anti-rabbit IgG (Biyun Tian, Inc.); Percoll lymphocyte isolate P8370 (Solarbio, Inc.); LPDCs magnetic bead sorting kit (OX62) (MiltenyiBiotec, Inc.) Anti-RatCD103, PE (Bioscience, Inc.); Rat spleen lymphocyte isolate LTS1083PK-200 (TBD, Inc.); CD4 Magnetic Bead Sorting Kit (MiltenyiBiotec, Inc.); CD4MonoclonalAntibody;CCK-8 Kit C0039 (Biotronix, Inc.); Rat Interleukin 12 (IL-12) ELISA Kit, Rat Interferon Gamma (IFN-γ) ELISA Kit, Rat Interleukin 4 (IL-4) ELISA Kit, Rat Interleukin 9 (IL-9) kit, Rat Interleukin 6 (IL-6) ELISA kit, Rat Interleukin 17A (IL-17A) ELISA kit, Rat Transforming Growth Factor β (TGF-β) ELISA kit (Jiangsu Jingmei, Inc.).

### 2.3 Experimental methodology

#### 2.3.1 Grouping of animals

Rats were randomly divided into 3 groups of 15 rats each using random number table method: Normal control group (NC n=15); IBS-D model group (Model n=15); MMF intervention IBS-D model group (MMF n=15). The reasonableness of the sample size was evaluated through degrees of freedom.

#### 2.3.2 IBS-D visceral hypersensitive animal model establishment

The IBS-D visceral hypersensitivity model was established by acetic acid enema combined with restraint stress in each model group and MMF intervention group (La et al., 2003; Bourdu et al., 2005; Li et al., 2015; Zhuang et al., 2022; Zhuang et al., 2024). The basic environment of the animal house was set at room temperature of 22-24 °C, humidity <60%, and noise <50 db, maintaining a daily 12-hour day-12-hour night alternation. The experimental animals were guaranteed to drink and eat freely after entering the clean-grade animal house, and the animals were acclimatized for one week and then used for subsequent experimental studies.

##### 2.3.2.1 NC group

On the 1st day of the experiment, rats were treated with 1 mL of 0.9% saline enema, and then put back to the cage to move freely after the end of the experiment, and then treated with 1 mL of 0.9% saline by gavage on the 10th day of the experiment. On the 10th day of the experiment, the rats were treated with 1mL of 0.9% saline by gavage. The gavage treatment was continued for 7 days.

##### 2.3.2.2 Model group

On the first day of the experiment, rats were given an enema of 1mL of 4% acetic acid solution, followed by rinsing with 1mL of 0.01mol/L PBS solution. After the procedure, they were returned to the cage for free activity. On the 7^th^ day of the experiment, they were subjected to restraint stress for 3 days. Starting from the 10th day of the experiment, they were given 1mL of 0.9% saline solution by gavage for 7 days.

##### 2.3.2.3 MMF group

On the 1st day of the experiment, the rats were irrigated with 1 mL of 4% concentration acetic acid, followed by a 1mL rinse with 0.01mol/L PBS, followed by free movement in the cage, on the 7^th^ day of the experiment, the restraint stress was performed for 3 days, and on the 10th day of the experiment, the MMF granules were prepared as an aqueous solvent with a raw drug content of 0.4 g/ml and administered to rats by gavage at a dose of 2 g/kg for 7 days.

#### 2.3.3 Model evaluation

Monitor the General condition of rats: The bowel movements, water and meal intake, nutritional status and hair color, activity.

Using the abdominal withdrawal reflex (AWR) scoring system to evaluate the modeling effect of visceral hypersensitivity in IBS-D rat model on the 17^th^ day (Zhuang et al., 2022; Al-Chaer et al., 2000).

#### 2.3.4 Anesthesia and execution of animals

Blood was collected from the abdominal aortic after the rats anesthetized by intraperitoneal injection of 3% pentobarbital (25 mg/kg) on the 19^th^ day; Tissue specimens were collected after the rats were finally executed by cervical dislocation on the 19^th^ day.

#### 2.3.5 Specimen collection

Fecal samples (It consists of fresh feces of the last 4 hours on the 19^th^ day, the lumen contents of the colon collected after PBS flushing, and colon mucosal tissue obtained by gentle scraping with a cover glass); Abdominal aortic blood; Colon tissue specimen; Spleen specimen.

#### 2.3.6 Detection method

Fluorescent quantitative PCR (qPCR) was used to detect the quantities of Bifidobacterium, Lactobacillus, and Escherichia coli; Fluorescein isothiocyanate-dextran (FITC-Dextran) was used to assess intestinal permeability; Immunofluorescence double labeling was used to detect the colocalization of the main structure of flagellin protein FliC and TLR5 in colonic mucosa; Immunohistochemistry was used to examine the expression of target proteins in the colonic mucosa of each group of rats; Immunomagnetic bead separation (MACS) was used to isolate colonic LPDCs and splenic CD4+ T lymphocytes; qPCR was used to detect the mRNA expression of TLR5, TRIF, and EPK1/2 in LPDCs; Cell Counting Kit (CCK8) was used to assess the proliferation of CD4+ T cells in the co-culture system of LPDCs and CD4+ T lymphocytes; Enzyme-linked immunosorbent assay (ELISA) was used to measure the levels of cytokines IL-12, IFN-γ, IL-4, IL-9, IL-6, IL-17A, TGF-β, and IL-10 in the supernatant from co-cultures of each group.

The above detection methods were performed according to the instructions of the reagent kits.

#### 2.3.7 Statistical methods

Measurement data were expressed as mean ± standard deviation (mean ± standard ± S), and count data were expressed as frequency (n) and percentage (%). Data were tested for Normality and Lognormality Tests by Shapiro-Wilk test, then selected for within-group comparisons using one-way multi-sample analysis of variance (ANOVA) if it follows a normal distribution, if not multi-sample rank-sum test (Kruskal-Wallis) will appropriate, and two-by-two comparisons between groups were made using Tukey method acted on when there were within-group differences, and immunofluorescence was performed using ImageJ software, Immunohistochemistry pictures were semi-quantitatively counted using GraphpadPrism software, and the data were statistically analyzed using GraphpadPrism 9.5.1 and Figdraw software to make graphs, and a statistical difference was considered to be statistically significant at P < 0.05.

## 3 Results

### 3.1 General condition of rats

The rats in model group showed increased proportion of loose watery or soft stools, perianal contamination with fecal residue, increased water intake, decreased food intake behaviors after acetic acid gavage modeling, and showed significantly reduced autonomous activities, easily provoked by peers, and irritable behaviors after restraint stress, in which the rats in the MMF group showed reduced autonomous water intake and increased rodent behavior during the period of gavage. None of the above abnormal behaviors were observed in the normal control group of rats.

### 3.2 Visceral sensitivity in rats

Observe the defecation of rats in each group before and after the experiment, and record the defecation status from 9:00 to 10:00 am on the 17th day of the experiment to assess the changes in gastrointestinal motility of rats. Calculate and compare the abdominal withdrawal reflex (AWR) scores of rats to evaluate changes in gastrointestinal sensitivity. The results suggest:

#### 3.2.1 Intestinal motility in rats

After the IBS-D modeling, the frequency of soft and loose stools was significantly higher, while the frequency of hard stools was significantly lower in the Model group compared to the NC group (p<0.0001, p<0.0001, p<0.0001). The rats in the MMF group exhibited a significantly higher frequency of soft and loose stools, with no significant difference observed in the number of hard stools (p=0.2002, p=0.7940, p=0.0071).

When compared to the Model group, the MMF group exhibited a reduction in the frequency of soft and liquid stools, alongside an increase in the occurrence of hard stools in rats (p=0.0006, p=0.0001, p=0.0006).

**Figure 1:**
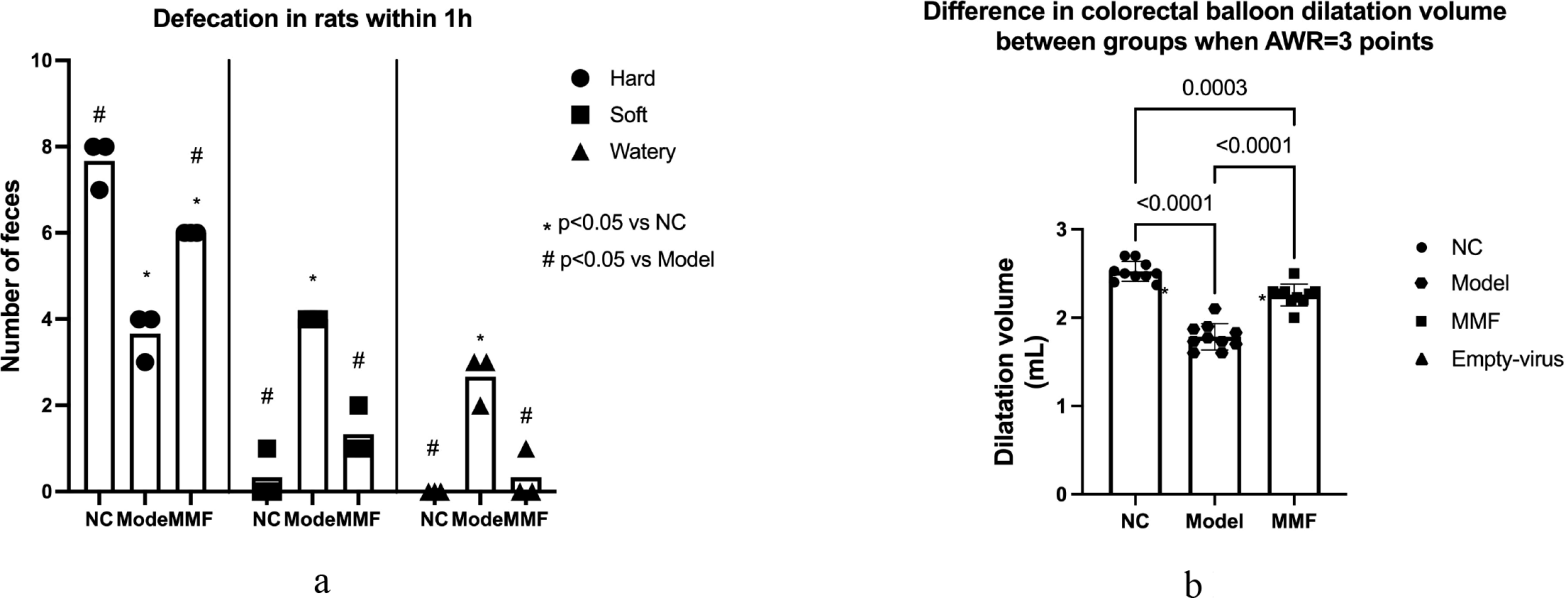
Comparison of visceral sensitivity among groups. **(a)** Defecation of rats in each group **(b)** Balloon dilatation volume in colorectum of rats in each group at AWR = 3.

This indicates that intestinal motility was significantly elevated in IBS-D group, and MMF can effectively alleviate intestinal hypermobility in IBS-D rats. For details, see **Figure 1a**.

#### 3.2.2 Colonic sensitivity in rats

After the IBS-D modeling, the balloon dilatation volume values at AWR = 3 in the colorectum of rats from both the Model and MMF groups were significantly lower than those observed in the NC group. This indicates that the sensitivity to colorectal pressure perception in rats from the Model group was markedly heightened (p<0.0001; p=0.0002).

In contrast, when compared to the Model group, it was demonstrated that the rats in the MMF group exhibited an increase in balloon dilatation volume and a decrease in colorectal sensitivity to intraluminal balloon pressure when AWR = 3. (p<0.0001).

The results indicate that the visceral sensitivity of rats increased following the modeling of IBS-D, which means the successful establishment of the model. And MMF was able to reduce the visceral hypersensitivity of rats with IBS-D, as illustrated in **Figure 1b**.

The comparison of visceral sensitivity include The defecation of rats in each group three repeated tests shows in **Figure 1a**, and the changes of balloon dilatation volume in colorectum of rats which shows in **Figure 1b**. (Adopt Tukey statistical method. Refer to the annotations in the reference image for grouping identification).

### 3.3 The content of *Lactobacillus, Bifidobacterium* and *Escherichia coli*

qPCR was used to quantify the fecal *Lactobacillus*, *Bifidobacterium*, and *Escherichia coli* content in each group of rats:

After the modeling of IBS-D, the counts of *Lactobacillus* and *Bifidobacterium* in the feces of rats from the Model and MMF groups were significantly lower compared to NC group (*Lactobacillus*: p<0.0001; *Bifidobacterium*: p<0.0001). In contrast, *Escherichia coli* was significantly upregulated in the Model group (p<0.0001), whereas no significant differential change was observed in the MMF group (p=0.3946). By comparing with the Model group, it was demonstrated that the fecal counts of *Lactobacillus* and *Bifidobacterium* were significantly elevated in the MMF group, whereas the counts of *Escherichia coli* were significantly reduced (*Lactobacillus*: p<0.0001; *Bifidobacterium*: p<0.0001; *Escherichia coli*: p<0.0001).

**Figure 2:**
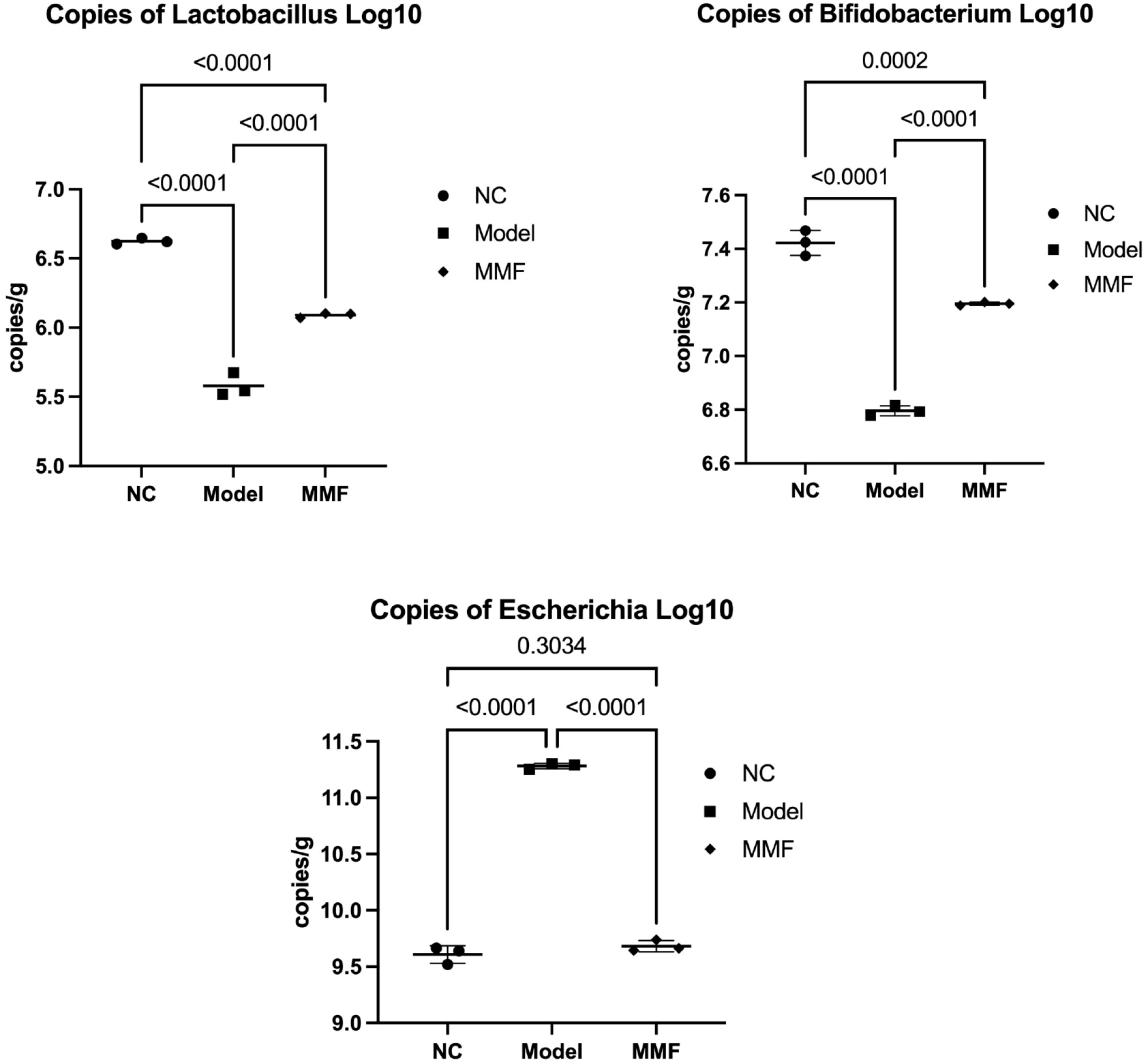
Counts of Lactobacillus, Bifidobacterium, and Escherichia coli in feces. qPCR was used to quantify the fecal Lactobacillus, Bifidobacterium, and Escherichia coli content in each group of rats three repeated tests. (Adopt Tukey statistical method. Refer to the annotations in the reference image for grouping identification.)

It demonstrated that the fecal microbial community of rats subjected to IBS-D modeling exhibited a reduction in the populations of *Lactobacillus* and *Bifidobacterium*, alongside an increase in the levels of *Escherichia coli*. While the MMF intervention led to a resurgence in the counts of fecal *Lactobacillus* and *Bifidobacterium*, while concurrently decreasing the fecal *Escherichia coli* counts in the IBS-D model. For further details, please refer to **Figure 2**.

### 3.4 Colonic permeability and the Flagellin-TLR5 expression in colonic mucosa

The intestinal permeability of rats in each group was assessed using FITC-Dextran. Additionally, laser confocal microscopy was employed to observe the localization of flagellin and TLR5 proteins, which were double-labeled through immunofluorescence in the colonic mucosa. In this analysis, TLR5 was represented by red fluorescence and flagellin by green fluorescence.

The results indicated that the serum levels of FITC-Dextran were significantly elevated in the Model group of rats when compared to the NC group (p<0.0001; p<0.0001). The expression of flagellin and TLR5 protein in the lamina propria of the colonic mucosa was also increased (all p<0.0001). In comparison to the Model group, there was a notable down-regulation of FITC-Dextran levels in the serum of rats in the MMF group (p<0.0001), along with a significant reduction in the expression of colonic mucosal flagellin and TLR5 protein (p all<0.0001). No significant differences were observed in the levels of colonic mucosal flagellin and TLR5 proteins when compared to the NC group (flagellin: p=0.9099; TLR5: p=0.9900).

**Figure 3:**
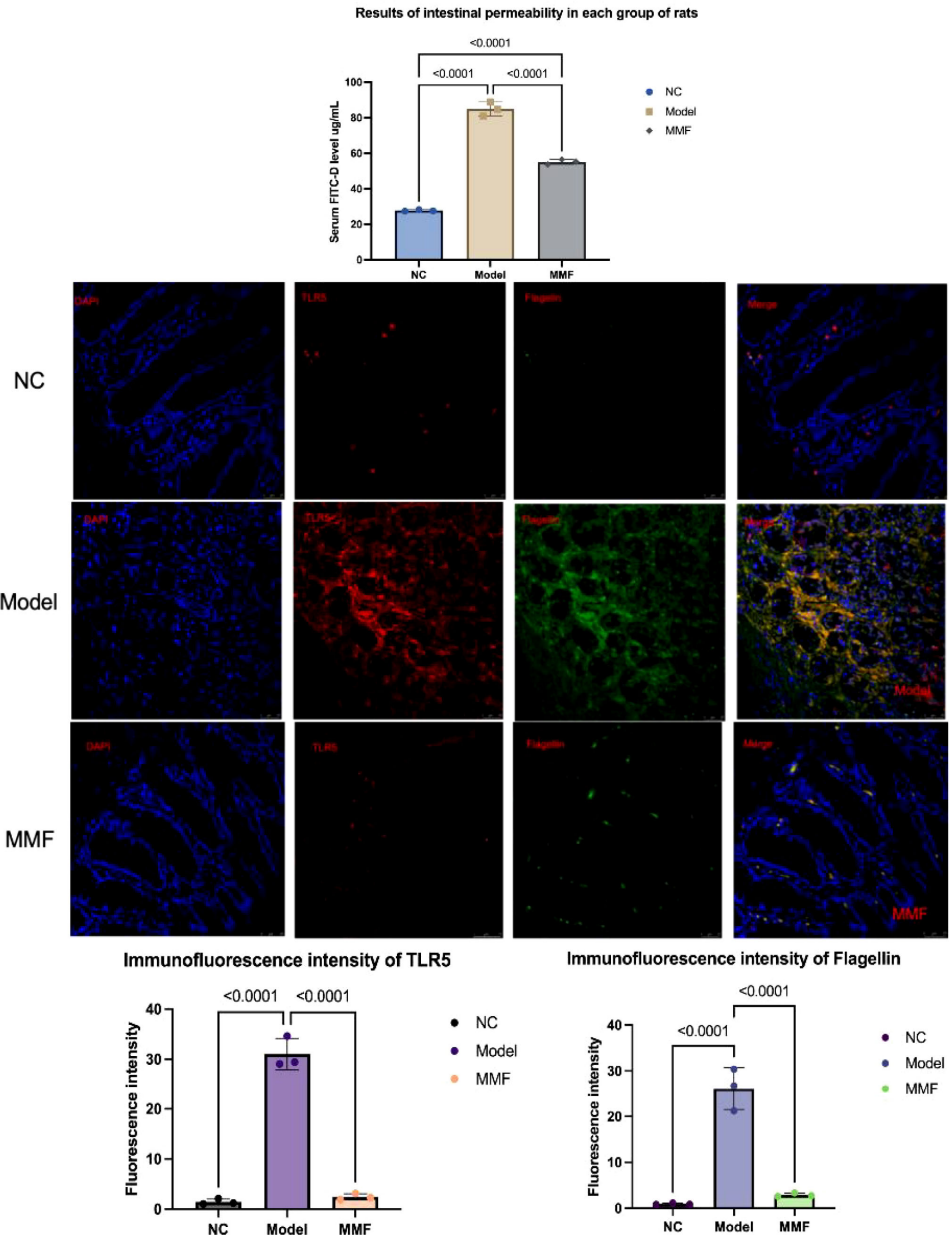
Intestinal permeability and expression of flagellin and TLR5 protein in the lamina propria of the mucosa of rats in various groups. FITC-Dextran was used to detect the intestinal permeability of rats and laser confocal microscopy was employed to observe the localization of flagellin and TLR5 proteins of colon three repeated tests. (Adopt Tukey statistical method. Refer to the annotations in the reference image for grouping identification.)

The aforementioned results indicated that the intestinal permeability of IBS-D rats was elevated, leading to an increased risk of intestinal bacterial translocation. A significant infiltration of bacterial flagellin was observed in the lamina propria of the mucosa, which resulted in a heightened expression of the flagellin-specific receptor TLR5 protein on the surface of the lamina propria dendritic cells (LPDCs). In IBS-D rats, intestinal permeability was restored through the intervention of MMF, and the expression levels of bacterial flagellin in the lamina propria, as well as TLR5 protein on the surface of LPDCs, were modulated. Please refer to **Figure 3**.

### 3.5 The expression of TLR5, TRIF, and p-EPK1/2 signals in LPDCs

Magnetic activated cell sorting (MACS) was used to isolate LPDCs from colon mucosa, and trypan blue staining showed cell activity>90%. The mRNA expression of TLR5, TRIF, and p-EPK1/2 in LPDCs was detected by qPCR. The expression of TLR5, TRIF, and p-EPK1/2 proteins in LPDCs of rats in each group was detected by WB, and the results showed that:

Compared with NC, it was found that TLR5, TRIF, p-EPK1/2 mRNA and protein contents in LPDCs in Model group and MMF group were increased (Model:p<0.0001, p<0.0001, p<0.0001, p<0.0001; MMF: p=0.0002, p<0.0001, p=0.0029, p<0.0001). Compared with Model group, the mRNA and protein contents of TLR5, TRIF and p-EPK1/2 in LPDCs in MMF group were significantly down-regulated (p<0.0001, p<0.0001, p<0.0001, p<0.0001).

**Figure 4:**
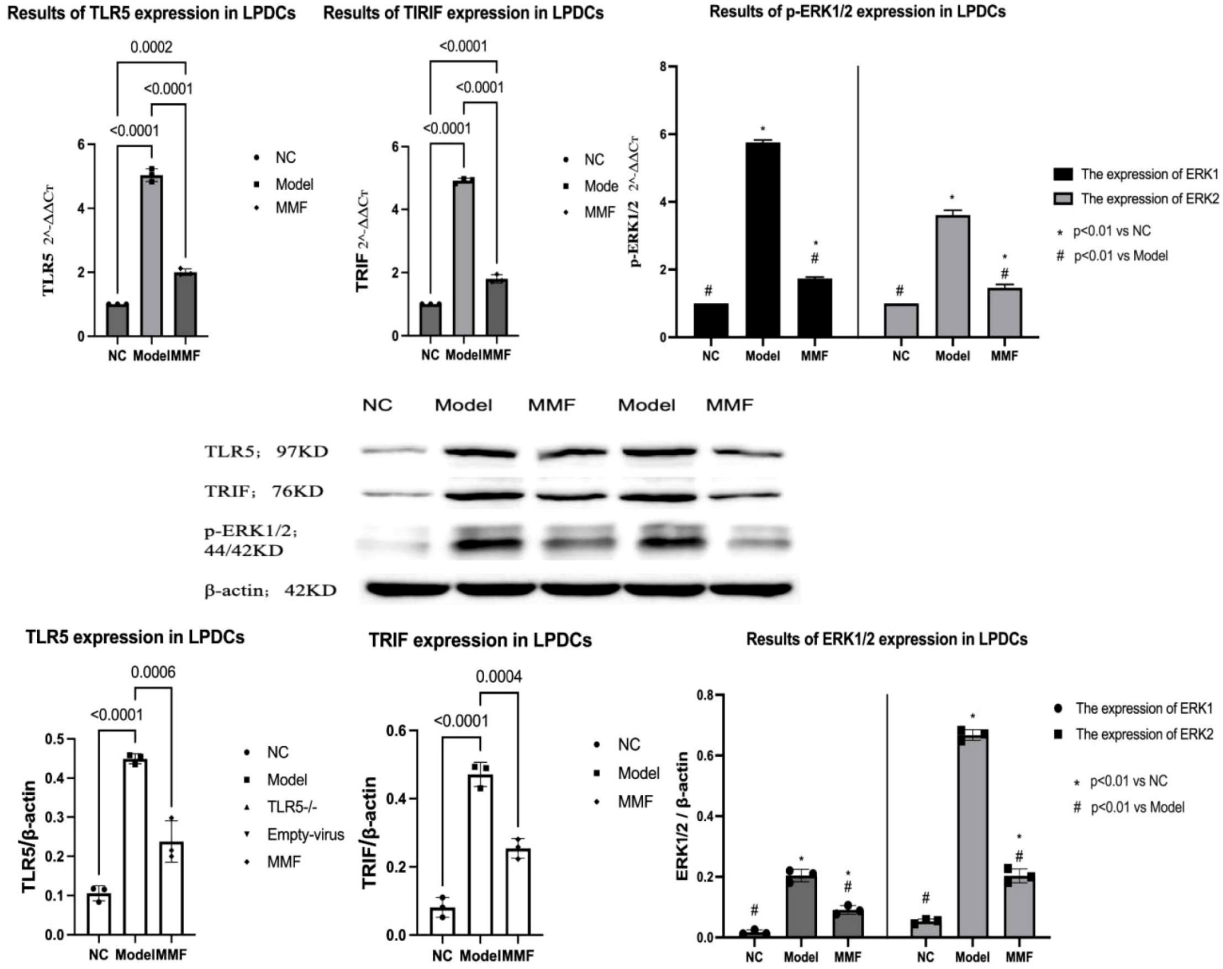
Expression of TLR5, TRIF and p-EPK1/2 in LPDCs of rats in each group. The mRNA expression of TLR5, TRIF, and p-EPK1/2 in LPDCs was detected by qPCR. The expression of TLR5, TRIF, and p-EPK1/2 proteins in LPDCs of rats in each group was detected by WB three repeated tests. (Adopt Tukey statistical method. Refer to the annotations in the reference image for grouping identification.)

It indicated that the expression of TLR5、TRIF、p-EPK1/2 were increased inside the LPDCs in IBS-D model, and the overexpression of proteins could be inhibited by the intervention of MMF. See **Figure 4** for details.

### 3.6 The changes in proliferative capacity of autologous CD4+ T lymphocytes and type of lymphocyte cytokine secretion promoted by LPDCs

Using MACS to isolate CD4+T lymphocytes from the spleen of rats, trypan blue staining showed cell activity>90%.

**Figure 5:**
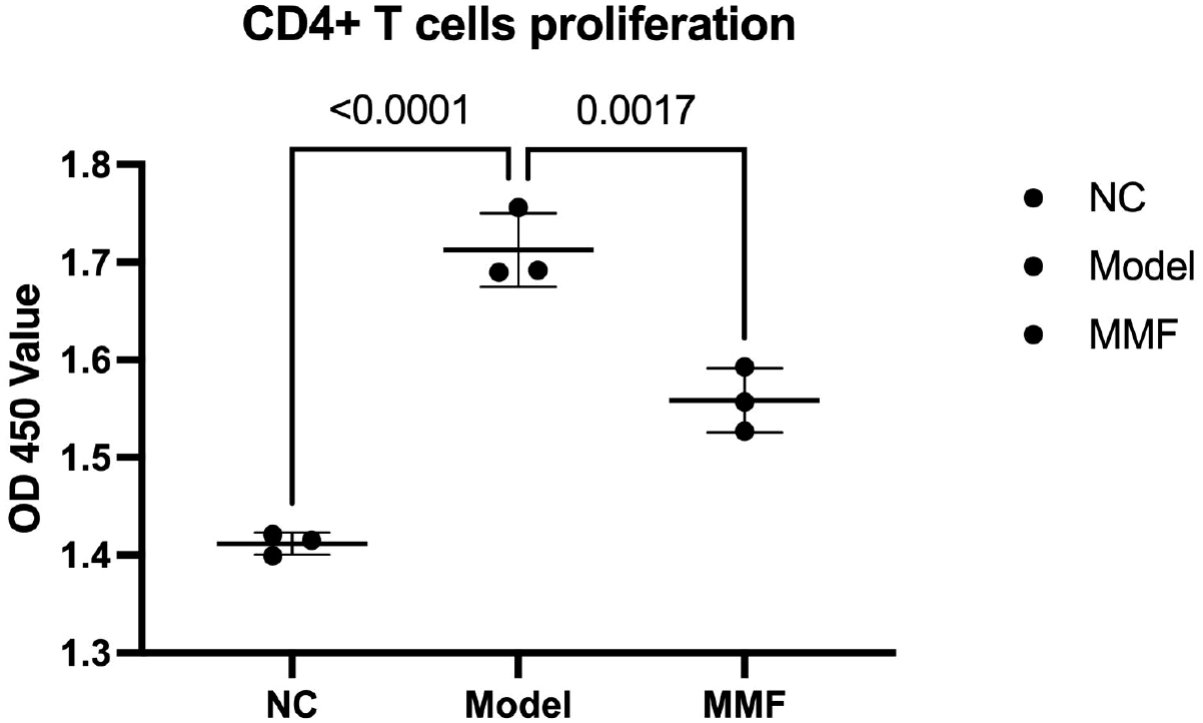
Ability of proliferation of CD4+ T lymphocytes promoted by LPDCs. The CCK-8 assay was used to detecte Lymphocyte proliferation ability for three times tests. (Adopt Tukey statistical method. Refer to the annotations in the reference image for grouping identification.)

1) Lymphocyte proliferation ability was detected by CCK-8 assay, and the results suggested that CD4+ T lymphocyte proliferation ability was enhanced in Model and MMF groups compared with NC (p<0.0001; p=0.0124). In contrast, the proliferative capacity of CD4+ T lymphocytes was significantly lower in the MMF group compared to the Model group (p=0.0091). See **Figure 5** for details.

The CCK-8 assay was used to detect Lymphocyte proliferation ability for three times tests. (Adopt Tukey statistical method. Refer to the annotations in the reference image for grouping identification).

2) ELISA was selected to detect the type and level of lymphocyte cytokine secretion in the co-cultured supernatant of cells.

Compared with the NC group, the secretion levels of IL-6, IL-17A, TGF-β, IL-12, IFN-γ and IL-4 were significantly higher. While the secretion level of IL-10, was significantly decreased (p<0.0001, p=0.0005, p<0.0001, p<0.0001, p<0.0001, p<0.0001, p<0.0001);

Compared with the Model group, IL-6, IL-17A, TGF-β, IL-12, and IL-4 secretion levels were significantly decreased in the MMF group (p<0.0001, p=0.0043, p<0.0001, p=0.0002, p=0.0002, p=0.0003), while IL-10 secretion levels were increased (p=0.0311).

**Figure 6:**
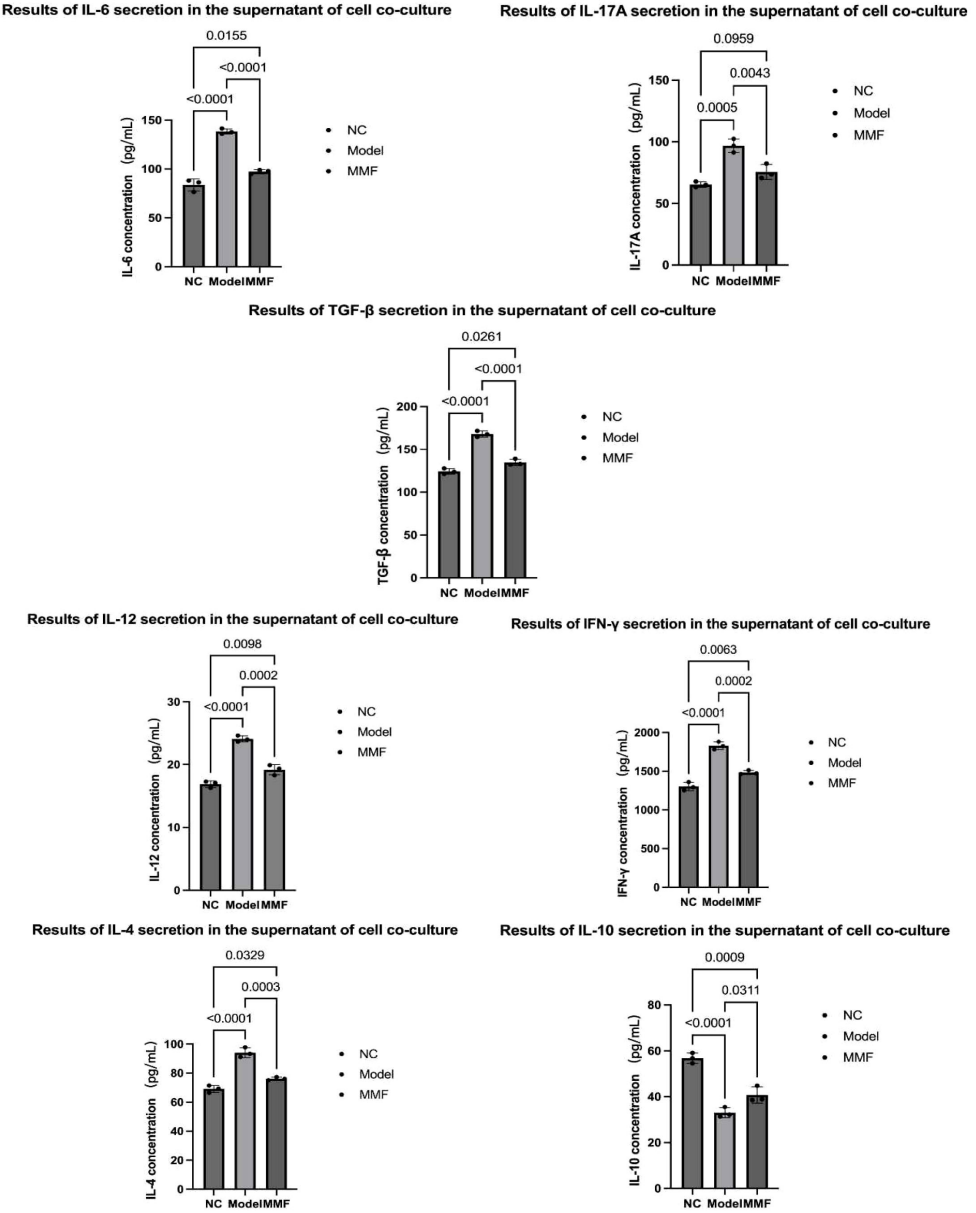
Changes of lymphocyte cytokine secretion promoted by LPDCs. ELISA was selected to detect the type and level of lymphocyte cytokine secretion in the co-cultured supernatant of cells for three times tests. (Adopt Tukey statistical method. Refer to the annotations in the reference image for grouping identification.)

The above results indicated that after IBS-D modeling, under the influence of LPDCs, the proliferative capacity of rat CD4+ T cells was hyperproliferative, and lymphocytes secreted more inflammatory mediators and immunostimulatory mediators (IL-6, IL-17A, TGF-β, IL-12, IL-4). In contrast, the ability to secrete IL-10, an immune homeostatic regulator, was diminished. In contrast, MMF intervention restored the above cytokine secretion imbalance, See **Figure 6** for details.

## 4 Discussion

In recent years, the significance of intestinal microbiota imbalance in the pathogenesis of IBS-D visceral hypersensitivity has gradually become evident. Relevant studies have indicated microbiological characteristics such as a reduction in the overall diversity of intestinal microecology, an overgrowth of conditionally pathogenic bacteria, a substantial decrease in the content of the dominant flora, and an increase in the proportion of harmful flora in patients with IBS-D (Mazzawi, 2022; Shrestha et al., 2022).

Furthermore, the imbalance in mucosal cytokine secretion resulting from an abnormal immune response in the intestinal mucosa has been established as a key factor in the development of visceral hypersensitivity in IBS-D. The activation of various lymphocyte, along with the prolonged infiltration of inflammatory mediators and immune-stimulating factors, can directly affect the intestinal submucosal and the spinal cord neurons which causing visceral hypersensitivity (Li et al., 2015; Jizhong et al., 2016; Lazaridis and Germanidis, 2018).

However, the correlation and pathogenesis between structural changes in microbiota and imbalance of cytokine secretion in the intestinal mucosa have not been elucidated.

Our results indicated that, consistent with the findings in patients with IBS-D, there was indeed an increase in intestinal colonization by *Escherichia coli* and a decrease in the number of *Lactobacillus* and *Bifidobacterium* in the IBS-D model. Additionally, we observed an increase in the permeability of the colonic mucosa and the presence of an infiltration of *Escherichia coli* flagellin in the intestinal mucosal lamina propria, inducing a higher expression of the flagellin-specific receptor, TLR5 on the LPDCs. activate the TRIF-ERK1/2 signal in LPDCs, promote the activation of LPDCs immune function, and enhance the proliferation of CD4+T lymphocytes. Lymphocytes secreted more inflammatory mediators and immune-stimulating mediators (IL-6, IL-17A, TGF-β, IL-12, IL-4), while the secretion of immune homeostatin regulator IL-10 was weakened, which mediated the abnormal immune response of intestinal mucosa and chronic low-grade inflammation in IBS-D visceral hypersensitive rats, resulting in the development of visceral hypersensitivity symptoms in IBS-D.

Simultaneously, our results finally shown that MMF can effectively mitigating the abnormal immune response and the chronic inflammation of the mucosa by FliC-TLR5-TRIF-ERK1/2 pathway. which alleviated the development of IBS-D visceral hypersensitivity successfully.

The *Escherichia coli* involved in this experiment has been demonstrated to enhance colonization in patients with IBS-D and in various animal models, as indicated by numerous studies (Mazzawi, 2022; Shrestha et al., 2022). Its distinctive flagellar structure can specifically bind to the intestinal host membrane protein receptor, Toll-like receptor 5 (TLR5), located on myeloid dendritic cells within the lamina propria of the intestinal mucosa via flagellin. This interaction enables *Escherichia coli* to play a significant role in the intestinal mucosal immune response to pathogenic bacteria as well as a crucial role in the chronic inflammatory response of the intestinal mucosa resulting from an imbalance of gut bacteria (Mohari et al., 2018).

TRIF protein, as a crucial adapter protein in the Toll-like receptor (TLR) signaling cascade, can activate ERK1/2 signaling via TLR5 when the intestinal mucosal barrier is compromised and when the colonization of harmful bacteria is increased. This activation induces the production of numerous chemokines and inflammatory mediators, such as interleukins and tumor necrosis factor, resulting in mucosal inflammatory cell infiltration (Choi et al., 2010; Zubair and Frieri, 2013; Bielaszewska et al., 2018). Concurrently, phosphorylated ERK1/2 can engage in the proliferation and differentiation of LPDCs, as well as facilitate the release of multiple immune mediators, which are pivotal in immune regulation (Guindi et al., 2018; Jiang et al., 2019).

Through our previous studies, we have demonstrated that the flagellin promotes the proliferation and maturation of the cellular phenotype of myeloid dendritic cells by FliC-TLR5-TRIF-ERK1/2 pathway, resulting in the enhancement of their immune functions and an improved ability to present antigens (Zhuang et al., 2024), which in turns mediate the differentiation of CD4+ T-lymphocytes, produce pro-inflammatory and immunostimulatory cytokines, and orchestrate mucosal inflammatory infiltration and immune responses (Strauch et al., 2010; Liu et al., 2016; Zhuang et al., 2016; Fucikova et al., 2019).

Currently, the role of abnormal intestinal mucosal immune responses and chronic inflammatory infiltration mediated by intestinal dendritic cells in the development of visceral hypersensitivity is garnering increasing attention in international studies. The intestinal mucosal immune profile is dysregulated in patients with IBS-D when compared to healthy controls (Berg et al., 2020). Furthermore, the mucosal immune system is abnormally activated, involving dendritic cells, lymphocytes, and the numerous cytokines they secrete, among which the imbalance in mucosal cytokine secretion has been demonstrated to be a direct contributor to visceral hypersensitivity (Kumar et al., 2022).

Inflammation-associated cytokines IL-17A, IL-6, and TGF-β were found to act directly on the intestinal mucosa due to their roles in mediating the inflammatory response and inducing inflammatory cell chemotaxis, resulting in an inflammatory infiltrate of the intestinal mucosa in IBS-D and correlating with the severity of visceral hypersensitivity symptoms (Ohman and Simrén, 2010; Vara et al., 2018; Shrestha et al., 2022).

The immunostimulatory factor IL-12 plays a crucial role in the low-grade inflammatory response of the intestinal mucosa in IBS-D by enhancing antigen presentation by macrophages and dendritic cells (Robinson, 2015; Kumar et al., 2022; Shrestha et al., 2022). Conversely, IFN-γ can disrupt the epithelial barrier function and tight junction proteins of the intestinal mucosa, thereby increasing intestinal permeability in IBS-D (Barbaro et al., 2016; Han et al., 2019). On the other hand, IL-4 promotes the activation and degranulation of mucosal mast cells through synergistic action with IL-9, which is implicated in the development of visceral hypersensitivity associated with IBS-D (Okayama et al., 2001; Fucikova et al., 2019; Zhuang et al., 2022).

However, IL-10, an important anti-inflammatory and immunomodulatory factor, can attenuate the antigen presentation of dendritic cells, which subsequently inhibits the secretion of inflammatory mediators by T-lymphocytes, thereby exerting an anti-inflammatory effect on the intestinal mucosa. Additionally, IL-10 inhibits the activation and degranulation of mast cells in conjunction with IL-4, which is vital for maintaining intestinal mucosal immune homeostasis (Gonsalkorale et al., 2003; Speiran et al., 2009). Consequently, the reduced secretion levels of IL-10 place the IBS-D mucosa at an increased risk for abnormal immune responses and chronic inflammatory infiltration (Shukla et al., 2018).

Modern pharmacological studies have demonstrated that MMF is effective in regulating the structure of intestinal flora and inhibiting the abundance of harmful bacterial in patients with IBS-D (Zhang et al., 2020). Additionally, it has been found to possess anti-inflammatory properties, repair intestinal mucosal epithelial cells, regulate intestinal wall muscle motility and ganglion function, and modulate intestinal mucosal immune function. It is often used in clinical practice for diarrheal diseases and has a relatively definite therapeutic effect. Studies have shown that numerous microorganisms are involved in the fermentation process of MMF, while by using ultra-high-performance liquid chromatography- tandem mass spectrometry and SEM analysis research indicates that the metabolites associated with these microorganisms, namely lipids, flavonoids, phenolic acids, lignins, coumarins, and organic acids, are the primary active components responsible for the medicinal effects of MMF, rather than the microorganisms themselves. We employ high-temperature stewing when using MMF, so the microbial communities present in the initial raw medicine cannot serve as accurate research subjects, so this might be the reasons why MMF induces a unique microbiota state, rather than simply restoring a “healthy” configuration (Bose et al., 2012; Sun et al., 2018; Li et al., 2025).

## 5 Conclusion

MMF can alleviate the abnormal immune response and cytokine secretion imbalance in the colonic mucosa and relieve visceral hypersensitivity symptoms. Its target may be closely related to the FliC-TLR5-TRIF-ERK1/2 pathway.

While our studies have not mentioned how long the characteristic structural changes in intestinal bacteria caused by the use of MMF can persist, nor how long it takes for the normal structure to be fully restored, therefore, we believe that our further long-term follow-up studies can be conducted. Meanwhile, our experiment is also limited to the rat model of IBS manifestations, and there are limitations in studying the individual functions of each component in MMF compounds. Therefore, further identification of the specific effects of each compound is needed. We will also set up a high-quality, multicenter, double-blind clinical study to further explore the role of MMF in IBS patients.

## Data Availability

The original contributions presented in the study are included in the article/supplementary material. Further inquiries can be directed to the corresponding author.
